# Methylenetetrahydrofolate Reductase C677T Polymorphism and Susceptibility to Cervical Cancer and Cervical Intraepithelial Neoplasia: A Meta-Analysis

**DOI:** 10.1371/journal.pone.0046272

**Published:** 2012-09-28

**Authors:** Ya Li Luo, Ping Ye, Qiong Hua Zhang, Ting Ting Hu, Min Hong Luo, Mei Qing Li, Qing Chen

**Affiliations:** Department of Epidemiology, School of Public Health and Tropical Medicine, Southern Medical University, Guangzhou, China; MOE Key Laboratory of Environment and Health, School of Public Health, Tongji Medical College, Huazhong University of Science and Technology, China

## Abstract

**Background:**

A number of studies have explored the association between methyl enetetrahydrofolate reductase (MTHFR) C677T polymorphism and susceptibility to cervical cancer and cervical intraepithelial neoplasia (CIN). However, results remained controversial. To address this gap, we decided to conduct a meta-analysis of all available published studies.

**Methods:**

Electronic literature searches of the PubMed, EmBase and Medline databases were performed up to April 30, 2012. Fixed-effects or random-effects model was used to calculate the pooled ORs for different genetic models.

**Results:**

A total of 12 case-control studies were ultimately identified. No statistical correlation was found between C677T variants and cervical cancer for the overall population. However, subgroup analyses on the White women pointed to a significant protective effect for individuals heterozygous or homozygous for the T-allele (for CT vs. CC: OR = 0.72, 95% CI 0.59–0.88; for TT vs. CC: OR = 0.69, 95% CI = 0.49–0.97; for CT+TT vs. CC: OR = 0.71, 95% CI 0.59–0.86). C677T variants were associated with neither combined nor stratified CIN among the overall population.

**Conclusions:**

This meta-analysis suggests that White women with mutant C677T genotypes might have a lower risk of cervical cancer, yet lacking enough statistical robustness. Further investigations are needed to get more insight into the role of this polymorphism in cervical carcinogenesis.

## Introduction

Cervical cancer is second only to breast cancer as the most common malignancies in both incidence and mortality among women worldwide, accounting for over 471,000 new cases and 250,000 deaths globally each year [1], [2]. Cervical intraepithelial neoplasia (CIN) is estimated to have at least 600,000 new cases per year [3], making (pre)neoplastic cervical disease a major public health threat and heavy burden to the society, especially in some high prevalent countries, such as India [4], Korea [5] and America [6].

Epidemiologic observations have implicated that infection with certain oncogenic types of human papillomavirus (HPV) is a major cause of cervical neoplasia [7]. However, there are still tremendous inter-individual variations contribute to the cervical neoplastic process among women infected with HPV, indicating that HPV infection alone cannot be entirely to blame.

Aside from HPV infection, a variety of socio-economic factors that were not traditionally associated with sexually transmitted diseases have been identified, such as cigarette smoking [8] and micronutrient deficiencies including vitamin C [9] and folate [10]. Among which the roles of folate in human carcinogenesis and in the treatments of cancers have been extensively discussed. The impacts of red cell folate concentration on cervical neoplasia have also long been investigated and a hypothesis that women with lower red cell folate level were more possible to be associated with high-risk types of HPV infection or cervical carcinogenesis, have been generally established through case-control [11]–[13] or cross-sectional [14] designs, thus stimulating much scientific interests in the possible influence of polymorphisms in folate coenzymes on cervical lesions.

The C677T (rs1801133) is the most common missense mutation localized in the gene encoding methylenetetrahydrofolate reductase (MTHFR). To date, a number of studies have explored the association between C677T polymorphism and susceptibility of cervical cancer and CIN [15]–[26]. Controversial results, however, existed among the affected women. To the best of our knowledge, no confirmed conclusions have been drawn concerning this genetic association issue. To address this gap, we decided to conduct a meta-analysis of all available published studies.

## Methods

### Studies Identification

Eligible articles up to April 30, 2012 were identified by searching the electronic literature databases (PubMed, EmBase and Medline). The keywords and search strategies were used as follows: (“squamous intraepithelial lesion” OR “cervical intraepithelial neoplasia” OR “cervical cancer”) AND (“methylenetetrahydrofolate reductase” OR MTHFR). Reference lists of reviews or original articles on this topic were also scanned to ensure that additional pertinent but previously omitted articles were included in the selected processes. If overlapping data were presented in several publications, only the most recent, largest or complete study was included. No published language restrictions were set in this meta-analysis.

### Inclusion Criteria

Studies meeting the following criteria were included: (1) original case-control studies; (2) exploration of MTHFR C677T polymorphism and susceptibility to cervical cancer or CIN; (3) all genotype distributions were reported in both case and control group; (4) allelic distributions in the control group conformed to the Hardy-Weinberg equilibrium (HWE) [27].

### Data extraction

For each study, data were extracted by two independent authors: first author's name, year of publication, country, ethnicity, type of control subjects, stage of cervical neoplastic lesions, sample-size of case and control and distributions of every genotype. Once the data extraction was complete, unsettled disputes were required to resolve. If a consensus could not be reached, a third author was consulted and a final decision was made by the majority of the votes. Different races/ethnicities were categorized following the U.S. Office of Management and Budget (OMB) standards for collecting data on race and ethnicity (1997 revision) [28].

### Statistical methods

The goodness-of-fit *χ^2^* test was used to assess the deviation from HWE in controls, statistical significance was defined as *P*<0.05. The individual and summary estimates were obtained by calculating the crude odds ratios (ORs), as well as their 95% confidence interval (CI) and the corresponding *P* value (the *P* value being significant if <0.05). The pooled ORs were estimated for co-dominant model (CT vs. CC; TT vs. CC), dominant model (CT+TT vs. CC) and recessive model (TT vs. CC+CT).

Heterogeneity between studies was assessed by calculating the *Q* statistic with *r*−1 (*r* is the number of analyzed studies) degrees of freedom (*df*) [29]. The fixed-effects model (Mantel-Haenszel method) [30] was used to calculate the pooled ORs with *P*>0.10 for *Q* statistic. Otherwise, the random-effects model (DerSimonian-Laird method) was used [31].

Moreover, the Begg's funnel plot [32] and Egger's linear regression test [33] were employed to assess the possible publication bias. Sensitivity analyses were performed to see whether any exclusion of the studies could affect the initial results. Data were imported into STATA 10.0 (Stata Corp, College Station, Tex) to conduct all statistical analyses.

## Results

### Selection of the included studies

**Figure 1:**
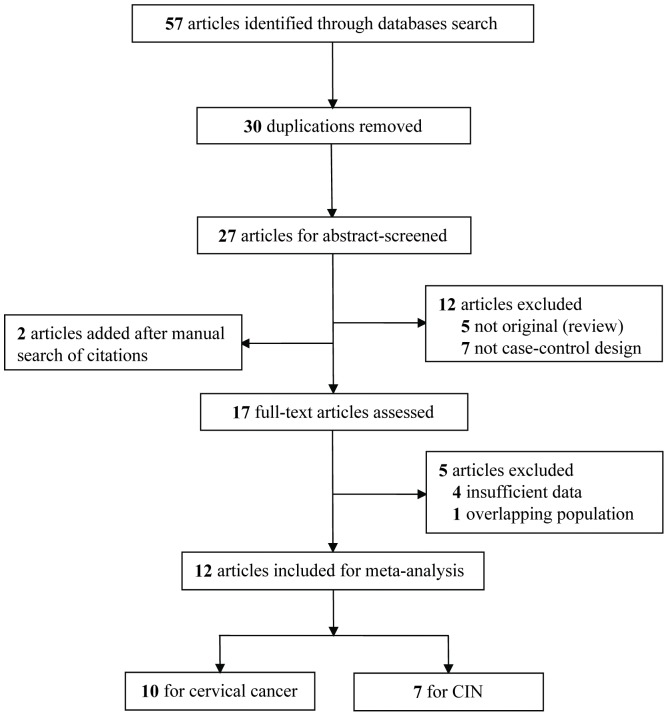
Flow chart explaining the selection of the 12 articles included in the meta-analysis.

This meta-analysis is guided by the PRISMA statement (**Protocol S1**). A number of 57 studies were preliminarily yielded based on the search terms. After abstract-screened and full-text assessed of these articles, a total of 12 articles met the inclusion criteria for detailed analysis (**Figure 1**, **Checklist S1**). The full list of 57 papers is available from the authors, on request.

### Description of the study characteristics

The included articles were all reported in English except for one in Spanish [21]. The majority of the 12 researches were conducted in European [17], [20], [24] and Asian [18], [19], [22], [23], [25] populations. Controls were derived from hospital-based participants except for Zoodsma et al. [20] and Mostowska et al. [24], where subjects were respectively recruited from a population-based organized cervical screening programme and unrelated healthy female volunteers who were from the same area of the cases. The DNA source for genotype determination was mainly from blood sample except for three studies [15], [17], [23], where cervical tissue was used. The selected characteristics of all included studies are described in **Table 1**.

**Table 1:** Characteristics of the studies of MTHFR C677T polymorphism and susceptibility to cervical neoplasia.

First Author	Year	Country	Ethnic Category	Control Type	Type of Cervical Neoplasia	DNA Source
Piyathilake [15]	2000	America	Mixed	HB	CIN I, CIN II/III	Exfoliated cervical cells (control), Cervical biopsy samples (case)
Goodman [16]	2001	America	Mixed	HB	CIN	Peripheral blood leukocytes
Lambropoulos [17]	2003	Greece	White	HB	CIN I, CIN II/III, Cervical Cancer	Exfoliated cervical cells, Fixed tumor materials (in 16 cancers)
Sull [18]	2004	Korea	Asian	HB	CIN I, CIN II/III, Cervical Cancer	Peripheral blood
Kang [19]	2005	Korea	Asian	HB	Cervical Cancer	Peripheral nucleated cells
Zoodsma [20]	2005	Netherlands	White	PB	CIN I, CIN II/III, cervical cancer	Blood serum
Delgado-Enciso [21]	2006	Mexico	White	HB	Cervical Cancer	Peripheral blood
Shekari [22]	2008	India	Asian	HB	Cervical Cancer	Peripheral blood
Kohaar [23]	2010	India	Asian	HB	CIN II/III, Cervical Cancer	Cervical scrapes (control), Fresh cervical biopsy samples (case)
Mostowska [24]	2011	Poland	White	PB	Cervical Cancer	Peripheral blood leucocytes
Prasad [25]	2011	India	Asian	HB	Cervical Cancer	Peripheral blood
Tong [26]	2011	Korea	Asian	HB	CIN I, CIN II/III, Cervical Cancer	Peripheral venous blood

HB: hospital-based, PB: population-based.

CIN: cervical intraepithelial neoplasia.

Mixed: population with individuals of different ethnicities.

Concerning cervical cancer, 10 studies were eligible with a total sample size of 1749 cases and 2451 controls. With respect to CIN, 7 studies were pooled for analysis (1223 cases and 2005 controls), all of which reported that CIN was histologically confirmed. The C677T genotype distributions in patients with cervical cancer or CIN and controls are summarized in **Table 2**, **Table 3**, respectively.

**Table 2:** The MTHFR C677T genotype distributions in controls and cervical cancer patients.

First Author	Year	Sample Size		Genotype Distributions						*P* value for HWE
				Control			Case			
		Control	Case	CC	CT	TT	CC	CT	TT	
Lambropoulos	2003	91	21	42	37	12	11	8	2	0.40
Sull	2004	454	246	153	221	80	73	115	58	0.99
Kang	2005	74	79	30	32	12	27	32	20	0.49
Zoodsma	2005	592	636	273	262	57	357	230	49	0.61
Delgado-Enciso	2006	89	70	20	49	20	18	34	18	0.34
Shekari	2008	200	200	125	68	7	170	28	2	0.54
Kohaar	2010	231	164	161	65	5	113	47	4	0.60
Mostowska	2011	168	124	69	81	18	56	59	9	0.42
Prasad	2011	125	63	116	8	1	57	6	0	0.06
Tong	2011	427	146	152	198	77	53	65	28	0.37

HWE: Hardy–Weinberg equilibrium for control group.

**Table 3:** The MTHFR C677T genotype distributions in controls and CIN patients.

CIN Categories	First Author	Year	Sample Size		Genotype distributions						*P* value for HWE
					Control			Case			
			Control	Case	CC	CT	TT	CC	CT	TT	
**Combined CIN**											
	Piyathilake	2000	31	64	16	12	3	17	36	11	0.74
	Goodman	2001	179	84	93	75	11	73	67	10	0.42
	Lambropoulos	2003	91	117	42	37	12	47	57	13	0.40
	Sull	2004	454	216	153	221	80	60	112	44	0.99
	Zoodsma	2005	592	318	273	262	57	148	141	29	0.61
	Kohaar	2010	231	39	161	65	5	28	11	0	0.60
	Tong	2011	427	319	152	198	77	106	156	57	0.37
**CIN I**											
	Piyathilake	2000	31	25	16	12	3	6	13	6	0.74
	Lambropoulos	2003	91	53	42	37	12	20	28	5	0.40
	Sull	2004	578	40	153	221	80	10	22	8	0.99
	Zoodsma	2005	592	54	273	262	57	27	21	6	0.61
	Tong	2011	427	159	152	198	77	52	82	25	0.37
**CIN II/III**											
	Piyathilake	2000	31	39	16	12	3	11	23	5	0.74
	Lambropoulos	2003	91	64	42	37	12	27	29	8	0.40
	Sull	2004	454	176	153	221	80	50	90	36	0.99
	Zoodsma	2005	592	264	273	262	57	121	120	23	0.61
	Kohaar	2010	231	39	161	65	5	28	11	0	0.60
	Tong	2011	427	160	152	198	77	54	74	32	0.37

HWE: Hardy–Weinberg equilibrium for control group, CIN: cervical intraepithelial neoplasia.

### Quantitative Synthesis

For all included studies, the allelic distributions of C677T in the control group were all consistent with HWE at the 0.05 level (**Table 2** **–** **3**), suggesting that obvious effects of natural selection and migration on genetic equilibrium had been avoided. The main results of the meta-analysis are outlined in **Table 4**.

**Table 4:** Results of meta-analysis for various genetic models of MTHFR C677T polymorphism.

Groups	Study (n)	Sample Size		CT vs. CC		TT vs. CC		CT+TT vs. CC		TT vs. CT+CC	
		Control	Case	*P_h_*	OR (95% CI)	*P_h_*	OR (95% CI)	*P_h_*	OR (95% CI)	*P_h_*	OR (95% CI)
Cervical Cancer											
Overall population	10	2451	1749	0.01	0.82 (0.63–1.06)	0.11	0.95 (0.76–1.19)	0.00	0.84 (0.64–1.11)	0.29	1.05 (0.85–1.28)
Ethnicity											
White	4	940	851	0.75	**0.72 (0.59–0.88)**	0.85	**0.69 (0.49–0.97)**	0.79	**0.71 (0.59–0.86)**	0.71	0.82 (0.60–1.12)
Asian	6	1511	898	0.00	0.86 (0.56–1.33)	0.22	1.23 (0.92–1.65)	0.00	0.90 (0.57–1.42)	0.39	1.25 (0.96–1.62)
Korean	3	955	471	0.85	1.04 (0.80–1.34)	0.44	1.37 (1.00–1.87)	0.63	1.13 (0.89–1.43)	0.51	1.34 (1.00–1.77)
Indian	3	556	427	0.00	0.73 (0.27–1.95)	0.28	0.54 (0.21–1.36)	0.00	0.71 (0.26–1.90)	0.41	0.60 (0.23–1.53)
CIN											
Combined CIN	7	2005	1223	0.49	1.15 (0.98–1.35)	0.66	1.14 (0.90–1.45)	0.35	1.14 (0.98–1.33)	0.92	1.04 (0.84–1.29)
CIN I	5	1595	331	0.34	1.25 (0.95–1.64)	0.37	1.14 (0.78–1.68)	0.27	1.22 (0.94–1.58)	0.54	0.99 (0.70–1.40)
CIN II/III	6	1826	742	0.58	1.13 (0.93–1.36)	0.80	1.15 (0.87–1.52)	0.50	1.13 (0.94–1.35)	0.94	1.07 (0.83–1.38)

*P_h_*: *P* values for heterogeneity from *Q*-test, CIN: cervical intraepithelial neoplasia.

Data in bold: statistical significance at 0.05 level.

No statistical significance was observed in C677T polymorphism and cervical cancer for the overall population at all genetic contrasts (CT vs. CC: OR = 0.82, 95% CI 0.63–1.06; TT vs. CC: OR = 0.95, 95% CI 0.76–1.19; CT+TT vs. CC: OR = 0.84, 95% CI 0.64–1.11; TT vs. CT+CC: OR = 1.05, 95% CI 0.85–1.28). Worth of note, however, significant heterogeneity between individual studies was seen in co-dominant model (CT vs. CC: *P_h_* = 0.01) and dominant model (CT+TT vs. CC: *P_h_* = 0.00), making stratified analyses necessary.

As White and Asian populations were involved in most studies, we also performed subgroup analyses to reduce the heterogeneity introduced by different ethnicity groups. The results for Asian population were replicated as non-significant association. When we further classified the Asian group according to certain countries, the Korean and Indian results continued to be null association, albeit with finite numbers of studies. As for the White population, the co-dominant model as well as dominant model turned out to be of statistical significance, with an OR of 0.72 (95% CI 0.59–0.88), 0.69 (95% CI 0.49–0.97) and 0.79 (95% CI 0.59–0.86), respectively, indicated a decreased cervical cancer risk for individuals heterozygous or homozygous for the T-allele among White women.

With respect to CIN, the pooled ORs did not show any statistical association between C677T polymorphism and CIN risk (CT vs. CC: OR = 1.15, 95% CI 0.98–1.35; TT vs. CC: OR = 1.14, 95% CI 0.90–1.45; CT+TT vs. CC: OR = 1.14, 95% CI 0.98–1.33; TT vs. CT+CC: OR = 1.04, 95% CI 0.84–1.29). As the CIN lesions could be divided into low and high grade lesions (CIN I and CIN II/III, respectively) and most of the individual studies have defined these two categorizations, data were available to perform a sub-analysis for CIN. Sound homogeneity was seen in two subgroups, and uncorrelated associations were also replicated (**Table 4**). The subgroup results based on ethnicity were not feasible for only limited papers provided the necessary data.

### Publication bias

Concerning cervical cancer, the shapes of the funnel plots did not reveal any sign of obvious asymmetry. Also, the results of Egger's test did not suggest any publication bias (CT vs. CC: *P* = 0.55; TT vs. CC: *P* = 0.54; CT+TT vs. CC: *P* = 0.60; TT vs. CT+CC: *P* = 0.36). Similarly, no significant publication bias was demonstrated regarding CIN (CT vs. CC: *P* = 0.11; TT vs. CC: *P* = 0.71; CT+TT vs. CC: *P* = 0.17; TT vs. CT+CC: *P* = 0.97).

### Sensitivity analyses

For the entire population, there was no remarkable departure from the initial ORs when the pooled estimates were recalculated by omitting one study at a time, and consistent non-significant association was observed across all genetic comparisons in cervical cancer and CIN studies, indicating that the overall findings was robust enough (data not shown).

For the White population, the sensitivity analyses pointed to a lower risk, as the total estimates documented, of the mutant genotypes, yet without statistically becoming a protection factor (for co-dominant model: *P* = 0.42 and 0.34, for dominant model: *P* = 0.33) when we excluded the Netherlands study [20], manifesting that this research had exerted a strong impact on the observed findings.

## Discussion

Methylenetetrahydrofolate reductase (MTHFR), a critical enzyme in folate-dependent metabolism of homocysteine, is involved in the conversion of 5,10-methylenetetrahydrofolate (5,10-methyleneTHF) to 5-methyltetrahydrofolate (5-methylTHF)–the primary circulating form of folate and the carbon donor for the remethylation of homocysteine into methionine [34]. C677T polymorphism, the most common functional single nucleotide polymorphism localized in MTHFR, is characterized by cytosine (C) to thymine (T) transition, which resulting in conversion from an alanine (GCC) to a valine (GTC) at codon 225 in the N-terminal catalytic domain of the protein. Compared to homozygous normal genotype (CC), both heterozygous (CT) and homozygous (TT) variants are shown to have increased enzyme thermolability, reduced MTHFR enzyme activity, elevated circulating homocysteine levels [34] and lower plasma and red blood cell folate concentrations [35]. Currently, it is convinced that folate deficiency is associated with carcinogenesis mainly through two mechanisms [36]: (1) The conversion of uracil to thymine, which is used for DNA synthesis and repair, requires methyl group provided by 5,10-methyleneTHF, therefore limited folate may interfere the thymidylate biosynthesis and subsequently lead to abnormal DNA synthesis, methylation and chromosome repair; (2) Low levels of 5-methylTHF cause DNA hypomethylation and potentially induce proto-oncogene expression as a consequence of depletion for cellular S-adenosylmethionine, which is also responsible for DNA methylation.

There is increasing interests in the investigations regarding associations of the MTHFR C677T polymorphism and susceptibility or resistance to cancer developments. However, results remain inconclusive, which impelled researchers to pay attention to this polymorphism at a meta-analytical level. On the whole, the protective effects of C677T polymorphism on colorectal cancer [37] and childhood acute lymphocytic leukemia [38] have been identified by two newly updated meta-analyses respectively included 61 and 21 published case-control studies. On the contrary, other large sample meta-analyses have proposed a greater risk in esophagus and gastric cancer [39] as well as breast cancer [40], and yet there were no evidence supporting that C677T variants contributed to lung cancer [41], [42], head and neck cancer [42] or prostate cancer [43] from currently available publications.

In reference to cervical disease susceptibility, the first study considering C677T polymorphism as a potential molecular marker was conducted by Piyathilake et al. [15] in 2000, which investigated 64 cases and 31 controls and suggested a 2.9-fold increased risk for CIN among women carrying either mutant heterozygous or homozygous genotype. Similar results were reported by Goodman et al. [16] who found women with at least one mutant T allele had a two-fold increased risk for cervical dysplasia with a larger sample-size. Lambropoulos et al. [17] firstly reported a null association between MTHFR polymorphism and risk of cervical cancer, and also, C677T variants were not related to the risk of CIN. Afterward, repeated researches from different regions emerged. However, either protective [20], [21], [22] or risk effects [18], [26] have been established, while in a few studies, null association was reported [19], [23], [24], [25].

There could be several factors ascribing to these contradicting findings. First of all, small numbers of study subjects were presented in some studies [15], [19], [21], which might lower the statistical power of the study by limiting the ability to estimate more precise association. Secondly, selection bias from study arm participation, both patients and controls, could be a possible explanation for discrepancies among individual studies, since all the women were recruited from different pools. Thirdly, variations during laboratory procedures such as DNA source (from cervical tissue or blood sample), use of commercial or self-design primer or PCR amplification condition might have affected the results. Furthermore, the genetic models applied in individual studies were largely diverse and generally only one or two models were used, thus incomprehensive or conflicting conclusions could be drawn by methodological difference. Last but not the least, the effects of genetic heterogeneity due to different ancestry of the study populations could not be ignored. The 677T allele frequencies, for example, have been reported more prevalent in Hispanics compared with non-Hispanics [44]. The heterogeneity that were inherent among subpopulations can lead to both type I and type II errors and confound the real association between C677T polymorphism and cervical neoplasia, where a positive or negative finding could be artificial inference attributable to population stratification.

To further clarify the relationship between C677T polymorphism and cervical disease, we performed this meta-analysis. The pooled ORs indicated that C677T variants were associated with neither combined nor stratified CIN among the overall population for all genetic models. There was either statistical significant correlation detected in the overall cervical cancer population, while subgroup analyses pointed to a decreased risk among white women with mutant genotypes. Notwithstanding, sensitivity analyses pointed to a lower risk, as the total results documented, but without statistically becoming a protective factor when the Netherlands study [20] was excluded. The above results were in accordance with most of the related studies as summarized in our meta-analysis and also, manifested that the role of MTHFR C677T polymorphism in cervical carcinogenesis development might be mediated by ethnicity.

We assumed that ethnicity differences, as we mentioned above, were the main reason for the inverse association driven by the White population. However, this finding was vulnerable to the statistical power in the sensitivity analyses. This lack of consensus might be resulted from two aspects. According to the U.S. OMB standards, the subgroup of White population was composed of only three European countries (Greece [17], Netherlands [20] and Poland [24]) and Mexico [21], the Netherlands represented the biggest proportion of the combined sample size (636/851 for case and 592/940 for control) and the only study that demonstrated a significant protective association among the four countries, thus it was probably that the significances were driven by this very large study. Moreover, only the Netherlands study was done in the setting of a population-based cervical screening aiming to detect cervical neoplasia susceptibility genes, therefore potential impacts might be introduced by the differences of study design. In light of the particularity of the Netherlands study, we considered the White population should be cautiously interpreted.

In this meta-analysis, we identified all studies in this field, and addressed the individual risk estimates as well as the pooled results using various genetic models. The accumulated data were substantial to overcome the issue proposed by Colhoun et al. [45] that conflicting results were primarily due to the small sample size. And the Begg's and Egger's tests did not detect any publication bias, indicating that our results were unbiased.

However, certain limitations in this study had to be acknowledged. First of all, large inter-study heterogeneity was observed, which meant interpretations of our findings should be undertaken carefully. The observed heterogeneity could be due to differences such as ethnicity variations, specified type of cervical cancer, selection criteria of case and control, socio-economic factors and so on. And yet sub-analyses on all these variables were not carried out as the study participants from previous studies varied a lot and data could not be presented in a uniform standard. Secondly, another polymorphism in linkage disequilibrium (LD), namely A1298C, which also caused decreasing MTHFR enzyme activity, though to a lesser extent [46], should also be considered to explain the effects of MTHFR polymorphism on cervical carcinogenesis alone or in combination with C677T genotypes. And yet our study was based on single-factor estimate. Moreover, given the complexity of tumor progress and the modest genetic effects from single gene, the environmental factors and random effects could not be ruled out. With regard to cervical diseases, individual behaviors, for example, age at first sexual intercourse [47], multiple sex partners [48], lack of barrier contraceptive use [49], were as well presented as risk factors, but interactions between these factors and C677T variants were not described in our study.

In summary, this meta-analysis suggests that White women with mutant C677T genotypes might have a lower risk of cervical cancer, yet lacking enough statistical robustness. Considering the limitation of this study, caution should be exercised in drawing any firm conclusions. Combined and comparative data sets from larger scale prospective studies are required to get more insight into the role of this polymorphism in the development of cervical carcinogenesis and to identify the joint effects with environmental factors.

## Supporting Information

Protocol S1 **PRISMA 2009 Checklist.**
(DOC)

Checklist S1 **Supplemental File for** **Figure 1** **.**
(DOC)
