# Supplementary material for: Methylenetetrahydrofolate Reductase C677T Polymorphism and Susceptibility to Cervical Cancer and Cervical Intraepithelial Neoplasia: A Meta-Analysis
Source: PLoS One. 2012 Sep 28;7(9):e46272. doi: 10.1371/journal.pone.0046272 (PMC3460879; doi:10.1371/journal.pone.0046272)
Supplement: Checklist S1 — Supplemental File for Figure 1 . (DOC) [file pone.0046272.s002.doc]

**Supplemental File for Figure 1**

1. **A number of 12 abstract-screened papers were excluded for the following reasons:**
2. **For not original (n=5):**

Eichholzer M, Luthy J, Moser U, Fowler B. (2001) Folate and the risk of colorectal, breast and cervix cancer: the epidemiological evidence. Swiss Med Wkly 131:539-549.

Nair S, Pillai MR. (2005) Human papillomavirus and disease mechanisms: relevance to oral and cervical cancers. Oral Dis 11:350-359.

Powers HJ. (2005) Interaction among folate, riboflavin, genotype, and cancer, with reference to colorectal and cervical cancer. J Nutr 135: 2960S-2966S.

Timar J, Nemzeti Onkologiai Kutatas-fejlesztesi Konzorcium. (2005) [Activity of the National Oncology R&D Consortium in 2004]. Magy Onkol 49:3-7.

Zoodsma M, Nolte IM, Te Meerman GJ, De Vries EG, Van der Zee AG. (2005) HLA genes and other candidate genes involved in susceptibility for (pre)neoplastic cervical disease. Int J Oncol 26:769-784.

1. **For not case-control design (n=7):**

Gerhard DS, Nguyen LT, Zhang ZY, Borecki IB, Coleman BI, et al. (2003) A relationship between methylenetetrahydrofolate reductase variants and the development of invasive cervical cancer. Gynecol Oncol 90:560-565.

(Using family-based transmission/disequilibrium test)

Henao OL, Piyathilake CJ, Waterbor JW, Funkhouser E, Johanning GL, et al. (2005) Women with polymorphisms of methylenetetrahydrofolate reductase (MTHFR) and methionine synthase (MS) are less likely to have cervical intraepithelial neoplasia (CIN) 2 or 3. Int J Cancer 113:991-997.

(Cohort design)

Chung HH, Kim MK, Kim JW, Park NH, Song YS, et al. (2006) XRCC1 R399Q polymorphism is associated with response to platinum-based neoadjuvant chemotherapy in bulky cervical cancer. Gynecol Oncol 103:1031-1037.

(Retrospective screening on patients)

Piyathilake CJ, Azrad M, Macaliso M, Johanning GL, Cornwell PE, et al. (2007) Protective association of MTHFR polymorphism on cervical intraepithelial neoplasia is modified by riboflavin status. Nutrition 23: 229-235.

(Cohort design)

Kim K, Kang SB, Chung HH, Kim JW, Park NH, et al. (2008) XRCC1 Arginine194Tryptophan and GGH-401Cytosine/Thymine polymorphisms are associated with response to platinum-based neoadjuvant chemotherapy in cervical cancer. Gynecol Oncol 111:509-515.

(Retrospective review on patients)

von Keyserling H, Bergmann T, Schuetz M, Schiller U, Stanke J, et al. (2011) Analysis of 4 Single-Nucleotide Polymorphisms in Relation to Cervical Dysplasia and Cancer Development Using a High-Throughput Ligation-Detection Reaction Procedure. Int J Gynecol Cancer 21:1664-1671.

(Retrospective screening on patients)

Agodi A, Barchitta M, Valenti G, Marzagalli R, Frontini V, et al. (2011) Increase in the prevalence of the MTHFR 677 TT polymorphism in women born since 1959: potential implications for folate requirements. Eur J Clin Nutr 65:1302-1308.

(Cross-sectional design)

1. **A number of 5 full-text reviewed papers were excluded for the following reasons:**
2. **For insufficient data (n=4):**

**3 papers for CT and TT genotypes could not be specified:**

Nandan NK, Wajid S, Biswas S, Juneja SS, Rizvi M, et al. (2008) Allelic variations in 5, 10-methylenetetrahydrofolate reductase gene and susceptibility to cervical cancer in Indian women. Drug Metab Lett 2:18-22.

Agodi A, Barchitta M, Cipresso R, Marzagalli R, La Rosa N, et al. (2010) Distribution of p53, GST, and MTHFR polymorphisms and risk of cervical intraepithelial lesions in sicily. Int J Gynecol Cancer 20:141-146.

Ragasudha PN, Thulaseedharan JV, Wesley R, Jayaprakash PG, Lalitha P, et al. (2012) A Case-Control Nutrigenomic Study on the Synergistic Activity of Folate and Vitamin B12 in Cervical Cancer Progression. Nutr Cancer 64:550-558.

**1 paper for genotype distributions in case and control were unavailable:**

Rao GG, Kurien A, Gossett D, Griffith WF, Coleman RL, et al. (2006) A case-control study of methylenetetrahydrofolate reductase polymorphisms in cervical carcinogenesis. Gynecol Oncol 101:250-254.

1. **For overlapping population (n=1):**

Tong SY, Lee JM, Song ES, Lee KB, Kim MK, et al. (2010) The effects of polymorphisms in methylenetetrahydrofolate reductase (MTHFR), methionine synthase (MTR), and methionine synthase reductase (MTRR) on the risk of cervical intraepithelial neoplasia and cervical cancer in Korean women. Cancer Causes Control 21:23-30.

(Same data were presented in paper by Tong *et al*, 2011)
